# Dynamic Versus Static Cardiac Biomarker Assessment for Prognostic Stratification After Percutaneous Coronary Intervention: A Systematic Review

**DOI:** 10.7759/cureus.110382

**Published:** 2026-06-07

**Authors:** Nabila N Anika, Mohamed K Said, Hamza Al Balushi, Fnu Vanshika, Shivam Singla, Sunita Kumawat, Hafiz H Zubair, Naeem Aslam, Ghazanfar Ali, Ali Ahmed

**Affiliations:** 1 General Surgery, University of South Florida Health, Florida, USA; 2 Internal Medicine, Holy Family Red Crescent Medical College, Dhaka, BGD; 3 Internal Medicine, Mansoura University, Mansoura, EGY; 4 Internal Medicine, The First Hospital of Jilin University, Changchun, CHN; 5 Internal Medicine, Peoples University of Medical & Health Sciences for Women, Nawabshah, PAK; 6 Internal Medicine, TidalHealth Peninsula Regional, Salisbury, USA; 7 Internal Medicine, St. Francis Medical Center, Lynwood, USA; 8 Internal Medicine, Pilgrim Hospital Boston, Nottingham, GBR; 9 Internal Medicine, Nottingham University Hospitals NHS trust, Nottingham, GBR; 10 Orthopedic Surgery, Bahawal Victoria Hospital, Bahawalpur, PAK; 11 Internal Medicine, Medicare Hospital, Faisalabad, PAK; 12 Internal Medicine, Services Hospital Lahore, Lahore, PAK

**Keywords:** cardiac biomarkers, delta biomarker, major adverse cardiovascular events, myocardial injury, nt-probnp, percutaneous coronary intervention, prognostic value, risk stratification, serial measurement, troponin

## Abstract

Cardiac biomarkers are widely used for risk stratification in patients undergoing percutaneous coronary intervention (PCI), yet their optimal mode of assessment remains uncertain. This systematic review evaluates the prognostic significance of serial, delta, and peak periprocedural cardiac biomarker measurements compared with single baseline measurements. A comprehensive literature search of PubMed/MEDLINE, Scopus, and Web of Science identified nine cohort studies encompassing diverse PCI settings, including elective, primary, and chronic total occlusion interventions. Biomarkers assessed included high-sensitivity and conventional troponin, N-terminal pro-B-type natriuretic peptide (NT-proBNP), and creatine kinase-myocardial band (CK-MB), with varying measurement strategies such as baseline, peak, serial, and delta assessments. The findings demonstrate that baseline troponin levels consistently predict outcomes, whereas post-procedural elevations show variable and threshold-dependent prognostic value. In contrast, serial assessment of NT-proBNP appears to enhance prognostic discrimination and reflects underlying pathophysiologic processes such as ischemic burden and reperfusion dynamics. Considerable heterogeneity was observed in biomarker timing, thresholds, and outcome definitions, precluding quantitative synthesis. Overall, the evidence suggests that dynamic biomarker assessment, particularly for natriuretic peptides, may offer incremental value over static measurements, although its clinical integration requires further validation. These findings support a more nuanced, context-dependent approach to biomarker interpretation in PCI populations.

## Introduction and background

Percutaneous coronary intervention (PCI) has become a cornerstone in the management of coronary artery disease (CAD) across a broad spectrum of clinical presentations, ranging from stable angina to acute myocardial infarction [1]. Despite advances in procedural techniques and adjunctive pharmacotherapy, patients undergoing PCI remain at risk for adverse outcomes, including mortality, recurrent ischemic events, and heart failure. Accurate post-procedural risk stratification is therefore essential for guiding clinical decision-making, optimizing resource utilization, and tailoring follow-up strategies. In this context, cardiac biomarkers have long been integrated into clinical practice as objective indicators of myocardial injury and hemodynamic stress [2,3].

Among these biomarkers, cardiac troponins and natriuretic peptides have received particular attention because of their strong associations with myocardial necrosis and ventricular dysfunction, respectively. Elevated troponin levels following PCI are frequently observed and have been incorporated into consensus definitions of periprocedural myocardial injury and infarction [4,5]. However, the clinical significance of these elevations remains a subject of ongoing debate, as minor increases may reflect procedural factors without clear prognostic implications. Similarly, natriuretic peptides such as N-terminal pro-B-type natriuretic peptide (NT-proBNP) provide insight into myocardial stress and volume overload, but their role in the post-PCI setting has not been as clearly established as in heart failure populations [6,7].

Traditionally, the prognostic utility of cardiac biomarkers has been assessed using single measurements obtained either at baseline or after the procedure, often interpreted according to predefined threshold values. Although this approach offers simplicity, it may not fully capture the dynamic biological processes that occur during and after myocardial ischemia and reperfusion [8]. Emerging evidence suggests that serial measurements and temporal changes in biomarker levels may provide additional prognostic information by reflecting evolving pathophysiologic states such as reperfusion success, infarct size, and ventricular remodeling. Furthermore, differences in biomarker behavior across clinical contexts, including elective versus emergent PCI and varying levels of procedural complexity, raise important questions regarding the generalizability of existing paradigms [6].

In light of these considerations, the present systematic review aims to evaluate the prognostic significance of serial, delta, and peak periprocedural cardiac biomarker measurements in comparison with single baseline measurements in patients undergoing PCI. In addition, this review seeks to explore how these associations vary according to biomarker class, procedural setting, and clinical endpoints, with the goal of providing a more nuanced understanding of biomarker-based risk stratification in contemporary PCI practice.

## Review

Materials and methods

Study Design and Reporting Framework

This systematic review was conducted to evaluate the prognostic significance of serial, delta, and peak periprocedural cardiac biomarker measurements compared with single baseline measurements in patients undergoing percutaneous coronary intervention (PCI). The methodology was developed in accordance with the Preferred Reporting Items for Systematic Reviews and Meta-Analyses (PRISMA) guidelines [9]. The review protocol was not prospectively registered in PROSPERO because the study was designed as a focused qualitative synthesis of prognostic cohort studies, with substantial anticipated heterogeneity in biomarker type, timing of measurement, PCI setting, and outcome definitions. No patient-level data collection, interventional assignment, or quantitative meta-analysis was planned. Nevertheless, the review question, eligibility criteria, search strategy, data extraction framework, risk of bias assessment, and synthesis approach were defined before article selection to preserve methodological transparency and reduce selection bias. The review question was structured using the population, intervention, comparator, and outcome (PICO) framework [10], in which the population comprised adult patients undergoing PCI, the intervention included serial, delta, or peak biomarker assessment; the comparator was a single baseline biomarker measurement; and the outcomes included mortality, major adverse cardiovascular events (MACE), reperfusion indices, and cardiac remodeling.

Search Strategy and Data Sources

A comprehensive literature search was conducted in PubMed/MEDLINE, Scopus, and Web of Science for studies published from January 2005 to December 2024. This timeframe was selected to capture contemporary PCI practice, modern biomarker assays, and clinically relevant prognostic studies. Database-specific search terms were developed using combinations of MeSH terms, free-text keywords, truncations where applicable, and Boolean operators, including AND and OR. The main search terms and Boolean combinations used for each database are summarized in Table 1. Reference lists of relevant articles were also manually screened to identify additional eligible studies.

**Table 1:** Search terms and Boolean operators used across databases.

Database	Search terms and Boolean combinations
PubMed/MEDLINE	(“percutaneous coronary intervention” OR “PCI”) AND (“cardiac biomarker” OR “cardiac biomarkers” OR “troponin” OR “high-sensitivity troponin” OR “high-sensitivity cardiac troponin” OR “NT-proBNP” OR “N-terminal pro-B-type natriuretic peptide” OR “CK-MB” OR “creatine kinase-myocardial band”) AND (“serial” OR “dynamic” OR “delta” OR “peak” OR “baseline” OR “post-procedural”) AND (“prognosis” OR “prognostic” OR “mortality” OR “major adverse cardiovascular events” OR “MACE” OR “reperfusion” OR “remodeling”)
Scopus	(“percutaneous coronary intervention” OR “PCI”) AND (“cardiac biomarker” OR “cardiac biomarkers” OR “troponin” OR “high-sensitivity troponin” OR “NT-proBNP” OR “N-terminal pro-B-type natriuretic peptide” OR “CK-MB” OR “creatine kinase-myocardial band”) AND (“serial” OR “dynamic” OR “delta” OR “peak” OR “baseline” OR “post-procedural”) AND (“prognosis” OR “prognostic” OR “mortality” OR “major adverse cardiovascular events” OR “MACE” OR “reperfusion” OR “remodeling”)
Web of Science	(“percutaneous coronary intervention” OR “PCI”) AND (“cardiac biomarker” OR “cardiac biomarkers” OR “troponin” OR “high-sensitivity troponin” OR “NT-proBNP” OR “N-terminal pro-B-type natriuretic peptide” OR “CK-MB” OR “creatine kinase-myocardial band”) AND (“serial” OR “dynamic” OR “delta” OR “peak” OR “baseline” OR “post-procedural”) AND (“prognosis” OR “prognostic” OR “mortality” OR “major adverse cardiovascular events” OR “MACE” OR “reperfusion” OR “remodeling”)

Study Selection Process

All identified records were screened independently based on titles and abstracts, followed by full-text assessment for eligibility. Studies were included if they met predefined inclusion criteria aligned with the review objective. Discrepancies in study selection were resolved through discussion and consensus. The study selection process was conducted in accordance with PRISMA recommendations to ensure transparency and reproducibility.

Eligibility Criteria

Studies were eligible for inclusion if they were prospective or retrospective cohort studies evaluating adult patients undergoing PCI and reported on the prognostic value of cardiac biomarkers measured either serially, as a delta change, or as peak post-procedural values. Eligible studies were required to include clinically relevant outcomes such as all-cause mortality, cardiovascular mortality, major adverse cardiovascular events, or surrogate markers of reperfusion or remodeling. Studies assessing commonly used biomarkers, including high-sensitivity troponin, conventional troponin, NT-proBNP, or CK-MB, were included to allow comparative analysis across biomarker classes. Both elective and primary PCI settings, as well as specialized populations such as chronic total occlusion interventions, were considered to capture variability in the clinical context.

Exclusion criteria included studies involving pediatric populations, animal or preclinical research, narrative reviews, editorials, case reports, and studies lacking clearly defined outcomes or extractable prognostic data. Studies that evaluated biomarkers solely for diagnostic purposes without assessing prognostic outcomes were also excluded. Meta-analyses were not included as primary evidence but were considered during background synthesis where relevant.

Data Extraction

Data extraction was performed systematically using a standardized framework. Extracted variables included study design, population characteristics, PCI setting, biomarker type, timing, and method of biomarker assessment, comparator definitions, clinical outcomes, and key prognostic findings. Particular emphasis was placed on distinguishing between baseline, peak, and serial biomarker measurements to align with the objectives of the review.

Risk of Bias Assessment

The methodological quality of the included studies was assessed using the Quality In Prognosis Studies tool [11], which is appropriate for evaluating prognostic factor research. Each study was evaluated across domains, including study participation, attrition, prognostic factor measurement, outcome measurement, confounding, and statistical analysis. Studies were categorized as having low, moderate, or high risk of bias based on domain-level assessments.

Data Synthesis

Given the heterogeneity in study design, biomarker timing, definitions of elevation, and outcome reporting, a quantitative meta-analysis was not performed. Instead, a structured narrative synthesis was undertaken, focusing on identifying patterns across studies, comparing baseline versus dynamic biomarker strategies, and evaluating differences across biomarker classes and PCI settings. This approach allowed for a comprehensive interpretation of the available evidence while maintaining methodological rigor.

Results

Study Selection Process

**Figure 1:**
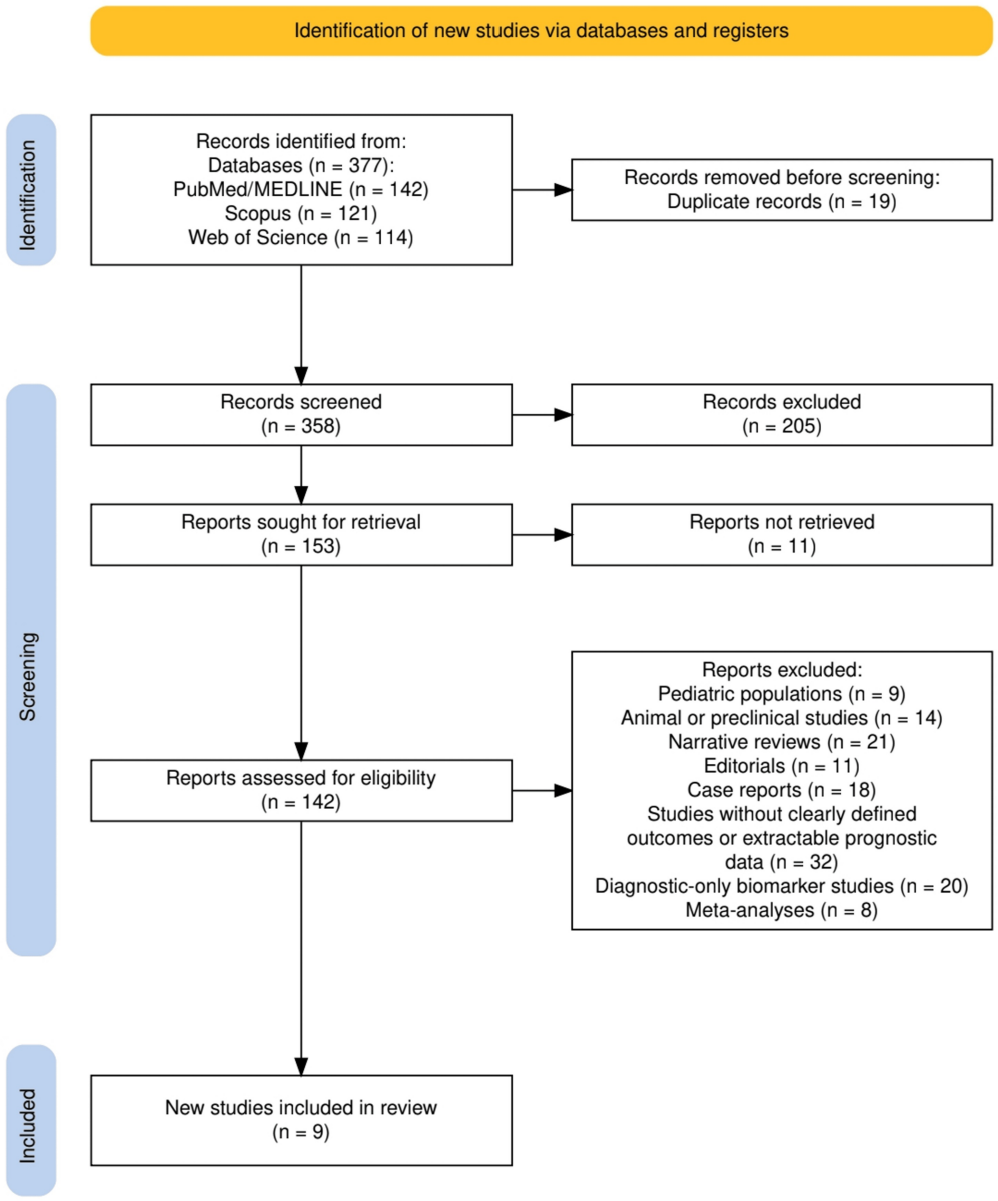
The PRISMA flow diagrma represents the study selection process. PRISMA: Preferred reporting items for systematic reviews and meta-analyses

The study selection process is illustrated in Figure 1 and was conducted in accordance with PRISMA guidelines. A total of 377 records were initially identified through database searching, after which 19 duplicate records were removed. The remaining 358 records underwent title and abstract screening, leading to the exclusion of 205 studies that did not meet the inclusion criteria. Subsequently, 153 full-text articles were sought for retrieval, of which 11 could not be accessed. A total of 142 studies were assessed for eligibility through full-text review. Of these, 133 studies were excluded for specific reasons, including pediatric populations, preclinical studies, narrative reviews, editorials, case reports, lack of clearly defined outcomes or extractable prognostic data, diagnostic-only focus, and meta-analyses. Ultimately, nine studies met all eligibility criteria and were included in the final qualitative synthesis.

Characteristics of the Selected Studies

The characteristics of the included studies are summarized in Table 2. A total of nine cohort studies were included, encompassing a broad range of PCI settings, including elective procedures, primary PCI in acute myocardial infarction, and high-risk interventions such as chronic total occlusion PCI. Sample sizes varied considerably, from smaller single-center cohorts to large multicenter populations, reflecting diverse clinical contexts and risk profiles. The studies evaluated multiple biomarker classes, including high-sensitivity and conventional troponin, NT-proBNP, and CK-MB, with differing approaches to measurement, such as baseline, peak post-procedural, serial, and delta assessments. Clinical outcomes were consistently centered on prognostic endpoints, including all-cause mortality, cardiovascular mortality, major adverse cardiovascular events, and, in some cases, measures of reperfusion and cardiac remodeling. Overall, the included studies demonstrate substantial heterogeneity in biomarker timing, thresholds, and outcome definitions, which informed the decision to undertake a narrative synthesis rather than quantitative pooling.

**Table 2:** Summary of included cohort studies evaluating static and dynamic cardiac biomarker assessment for prognostic stratification after percutaneous coronary intervention. PCI: Percutaneous coronary intervention, hs-TnT: High-sensitivity troponin T, hs-cTnT: High-sensitivity cardiac troponin T, cTnI: Cardiac troponin I, CK-MB: Creatine kinase–myocardial band, NT-proBNP: N-terminal pro–B-type natriuretic peptide, MACE: Major adverse cardiovascular events, AMI: Acute myocardial infarction, ACS: Acute coronary syndrome, CTO: Chronic total occlusion, URL: Upper reference limit, GRACE: Global Registry of Acute Coronary Events, ZRS: Zwolle Risk Score

Study (Year)	Design / Setting	Population (n)	PCI Type	Biomarker(s)	Biomarker Assessment (Timing/Type)	Comparator	Outcomes	Key Findings
Ndrepepa et al., 2016 [12]	Prospective cohort	5,626	Elective PCI	hs-TnT	Baseline + peak post-PCI	Baseline vs post-PCI	All-cause mortality (3-year)	Baseline hs-TnT predicted mortality; post-PCI levels did not add prognostic value
Zhou et al., 2022 [13]	Prospective cohort	3,249	Elective PCI	hs-cTnT	Peak post-PCI (threshold-based)	Baseline (normal)	MACE	Only elevations >8× URL predicted MACE; lower thresholds not significant
Ferreira et al., 2017 [14]	Retrospective cohort	407	Elective PCI	cTnI	Peak post-PCI (threshold-based)	None	All-cause mortality (1-year)	Troponin elevation associated with increased mortality; ≥5× URL most predictive
Koskinas et al., 2018 [15]	Prospective cohort	8,140	Elective PCI	hs-cTnT	Serial + delta + peak (threshold-based)	Baseline-adjusted	All-cause mortality (1-year)	Multiple thresholds predicted mortality; ~10× URL optimal balance
Goliasch et al., 2019 [16]	Retrospective cohort	3,712	CTO PCI	cTnT	Serial post-PCI (6–24 h, threshold-based)	Threshold-based	All-cause mortality	≥18× URL associated with increased mortality
Song et al., 2021 [17]	Retrospective cohort	2,616	CTO PCI	CK-MB, cTnI	Serial post-PCI (peak, threshold-based)	Definition-based	Cardiovascular mortality (5-year)	CK-MB predicted mortality; cTnI did not
Li et al., 2023 [18]	Prospective cohort	1,105	Primary PCI (AMI)	NT-proBNP	Serial (days 0–7, dynamic/peak)	Serial vs baseline + GRACE	MACE, mortality (3-year)	Serial NT-proBNP improved prognostic accuracy and risk stratification
Buchner et al., 2010 [19]	Prospective cohort	133	PCI (ACS)	NT-proBNP	Serial (0–96 h, dynamic/peak)	Serial vs baseline	Reperfusion, prognosis	Dynamic NT-proBNP reflected ischemia and reperfusion; 96 h levels predicted outcomes
Schellings et al., 2017 [20]	Prospective cohort	845	Primary PCI	NT-proBNP	Serial + delta (baseline, 18–24 h, 72–96 h)	Serial/delta vs baseline + ZRS	Mortality, MACE	18–24 h NT-proBNP and delta improved prognostic accuracy and early discharge stratification

Quality Assessment

The risk of bias assessment for the included studies is summarized in Table 3 and was conducted using the QUIPS tool [11], which is appropriate for prognostic factor research. Overall, most studies were judged to have a low risk of bias, particularly in the domains of prognostic factor measurement and outcome assessment, as biomarker assays and clinical endpoints such as mortality and major adverse cardiovascular events were generally well defined and objectively measured. The most common source of bias across studies was in the confounding domain, where a moderate risk was identified due to the potential influence of baseline disease severity, procedural complexity, and comorbid conditions that may not have been fully adjusted for in all analyses. A minority of studies demonstrated moderate overall risk of bias, primarily due to retrospective design, smaller sample size, or heterogeneity in biomarker measurement and reporting. Despite these limitations, the overall methodological quality of the included evidence was considered acceptable for qualitative synthesis.

**Table 3:** Risk of bias assessment of included prognostic cohort studies using the QUIPS tool. QUIPS: Quality In Prognosis Studies tool, PCI: Percutaneous coronary intervention, MACE: Major adverse cardiovascular events, AMI: Acute myocardial infarction

Study (Year)	Study type in this review	Suitable RoB tool	Study participation	Study attrition	Prognostic factor measurement	Outcome measurement	Study confounding	Statistical analysis and reporting	Overall risk of bias
Ndrepepa et al., 2016 [12]	Prospective cohort	QUIPS	Low	Low	Low	Low	Moderate	Low	Low
Zhou et al., 2022 [13]	Prospective cohort	QUIPS	Low	Low	Low	Low	Moderate	Low	Low
Ferreira et al., 2017 [14]	Retrospective cohort	QUIPS	Moderate	Moderate	Moderate	Low	Moderate	Moderate	Moderate
Koskinas et al., 2018 [15]	Prospective cohort	QUIPS	Low	Low	Low	Low	Moderate	Low	Low
Goliasch et al., 2019 [16]	Retrospective cohort	QUIPS	Low	Low	Low	Low	Moderate	Low	Low
Song et al., 2021 [17]	Retrospective cohort	QUIPS	Low	Low	Moderate	Low	Moderate	Low	Moderate
Li et al., 2023 [18]	Prospective cohort	QUIPS	Low	Low	Low	Low	Moderate	Low	Low
Buchner et al., 2010 [19]	Prospective cohort	QUIPS	Moderate	Moderate	Low	Moderate	Moderate	Moderate	Moderate
Schellings et al., 2017 [20]	Prospective cohort	QUIPS	Low	Low	Low	Low	Moderate	Low	Low

Discussion

Overall Synthesis

Across the included studies, the prognostic value of cardiac biomarkers following percutaneous coronary intervention (PCI) appears to depend not only on their absolute levels but also on their temporal behavior, with important variation across biomarker classes and clinical settings. Evidence derived from elective PCI cohorts, such as those reported by Ndrepepa et al. [12] and Zhou et al. [13], suggests that baseline biomarker levels often carry substantial prognostic significance, whereas post-procedural elevations provide limited or context-dependent incremental value. In contrast, studies incorporating serial or dynamic assessment, including those by Li et al. [18], Buchner et al. [19], and Schellings et al. [20], demonstrate improved risk discrimination and closer alignment with underlying pathophysiologic processes such as ischemic burden and reperfusion. Furthermore, variability across PCI settings, including high-risk populations such as chronic total occlusion (CTO) cohorts reported by Goliasch et al. [16] and Song et al. [17], highlights that the clinical utility of biomarker strategies is not uniform. Taken together, these findings support a conceptual shift from reliance on single biomarker measurements toward a more integrated, context-sensitive approach that incorporates temporal dynamics and biomarker-specific characteristics in prognostic assessment after PCI.

Baseline vs. Post-Procedural Signal

A central finding across the included studies is the variable prognostic relevance of baseline compared with post-procedural biomarker elevations. Evidence from Ndrepepa et al. [12] demonstrates that baseline high-sensitivity troponin levels retain independent prognostic significance, whereas post-PCI elevations do not consistently provide incremental value. In contrast, findings from Zhou et al. [13] and Ferreira et al. [14] suggest that post-procedural troponin elevations may carry prognostic relevance, although this appears contingent upon the magnitude of elevation and underlying patient risk. Further refinement is provided by Koskinas et al. [15], who illustrate that only higher thresholds of biomarker elevation are associated with meaningful risk stratification, highlighting a clear threshold-dependent relationship. Collectively, these observations indicate that post-procedural myocardial injury is not uniformly prognostic and that its clinical significance is modulated by baseline biomarker status, the extent of elevation, and patient selection. Accordingly, troponin elevation after PCI may primarily reflect procedural myocardial injury but only translates into adverse prognostic implications when exceeding clinically meaningful thresholds.

Limitations of Threshold-Based Definitions

The current paradigm of defining periprocedural myocardial injury based on fixed biomarker thresholds is challenged by substantial heterogeneity observed across the included studies. Reported prognostic cut-offs vary widely, ranging from 5 times to 18 times the upper reference limit, as demonstrated in studies such as Zhou et al. [13], Koskinas et al. [15], and Goliasch et al. [16], with additional variability introduced by differences in biomarker type and PCI setting. This inconsistency reflects not only methodological variation but also underlying differences in patient populations and procedural complexity, including elective versus high-risk interventions such as chronic total occlusion PCI. Furthermore, studies such as Song et al. [17] highlight discordance between biomarkers, with CK-MB demonstrating prognostic significance while troponin did not, underscoring the lack of biological uniformity in threshold-based definitions. From a methodological perspective, these findings emphasize the trade-off between sensitivity and specificity inherent in selecting higher versus lower cutoffs and suggest that fixed thresholds may oversimplify a complex and context-dependent biological process. As such, reliance on uniform biomarker thresholds may limit accurate risk stratification and reduce the generalizability of current definitions across diverse clinical scenarios.

Dynamic Assessment

One of the most important signals emerging from this review is that serial and dynamic biomarker assessment may offer prognostic value beyond that provided by single measurements alone. This is most clearly supported by Li et al. [18], who demonstrated that sequential NT-proBNP measurements improved risk discrimination for major adverse cardiovascular events and mortality, including measurable gains in reclassification metrics such as the net reclassification improvement and integrated discrimination improvement. Similarly, Schellings et al. [20] showed that NT-proBNP measured at 18 to 24 hours after primary PCI offered stronger prognostic utility than baseline measurement and improved early discharge stratification when integrated with an established clinical score. Mechanistic support for this concept is provided by Buchner et al. [19], who showed that serial NT-proBNP trajectories reflect reperfusion success, ischemic burden, and later clinical prognosis, with specific time points such as 96 hours appearing particularly informative. Taken together, these findings suggest that dynamic biomarker trajectories convey both prognostic and pathophysiologic information, capturing not only whether myocardial stress is present but also how it evolves after revascularization, which may be more clinically informative than static measurement alone.

Biomarker-Specific Patterns

An important interpretive distinction across the included studies is the difference in prognostic behavior between injury-based biomarkers, such as troponin, and stress-related biomarkers, such as NT-proBNP. Troponin-based studies, including those by Ndrepepa et al. [12], Zhou et al. [13], and Koskinas et al. [15], generally suggest that prognostic relevance is highly dependent on baseline status and on the magnitude of post-procedural elevation, indicating that troponin primarily reflects myocardial injury and is most informative when interpreted within a threshold-based framework. By contrast, NT-proBNP studies by Li et al. [18], Buchner et al. [19], and Schellings et al. [20] demonstrate stronger value for serial assessment, with repeated measurements improving prognostic discrimination and better capturing the evolving physiologic response to ischemia and reperfusion. This contrast suggests that natriuretic peptides may provide a broader representation of ventricular stress and hemodynamic adaptation, whereas troponin is more narrowly tied to myocardial necrosis or injury burden. From a prognostic perspective, this implies that dynamic models may be particularly well suited to NT-proBNP, which appears to offer greater incremental refinement than injury-based biomarkers when measured across multiple time points.

Clinical Context

The prognostic utility of biomarker strategies after PCI also appears to vary substantially according to procedural setting and underlying baseline risk. In elective PCI cohorts, such as those reported by Ndrepepa et al. [12], Zhou et al. [13], and Ferreira et al. [14], event rates were generally lower, and the prognostic signal of post-procedural biomarker elevation was more limited or highly threshold dependent. In contrast, primary PCI studies, particularly those by Li et al. [18] and Schellings et al. [20], suggest that dynamic biomarker assessment is more clinically informative in acute myocardial infarction, where evolving myocardial stress and reperfusion biology are central to early risk stratification. Chronic total occlusion PCI represents a distinct high-risk subgroup, and findings from Goliasch et al. [16] and Song et al. [17] indicate that larger biomarker rises and stricter threshold-based definitions may carry greater prognostic relevance in this technically complex setting. These differences indicate that the value of a given biomarker strategy cannot be assumed to be uniform across all PCI populations. Rather, its clinical usefulness is shaped by procedural complexity, baseline disease severity, and the expected event profile of the treated population.

Integration and Clinical Implications

Existing literature has largely focused on defining periprocedural myocardial injury through fixed troponin thresholds and consensus-based criteria, with particular emphasis on frameworks such as those evaluated in studies like Koskinas et al. [15], Goliasch et al. [16], and Song et al. [17], which sought to refine prognostic cut-offs and validate existing definitions. However, this approach has often overlooked the potential value of temporal biomarker dynamics and has rarely incorporated comparative analyses across different biomarker classes. The present synthesis extends beyond this traditional paradigm by integrating evidence on static versus dynamic assessment, contrasting injury-based markers such as troponin with stress-related biomarkers such as NT-proBNP, and situating these findings within specific procedural contexts. From a clinical perspective, these findings suggest that routine post-PCI troponin measurement may have limited standalone value unless interpreted in relation to baseline levels and clinically meaningful thresholds, as highlighted by Ndrepepa et al. [12] and Zhou et al. [13]. In contrast, serial NT-proBNP assessment, as demonstrated by Li et al. [18] and Schellings et al. [20], may offer incremental benefit in risk stratification and could support clinical decision-making processes such as early discharge planning. While these observations do not support an immediate shift in standard practice, they suggest that incorporation of dynamic biomarker strategies into existing risk models may enhance prognostic precision and warrant further prospective validation.

Strengths, Limitations, and Future Directions

This review has several notable strengths, including a focused and clinically relevant research question centered on dynamic versus static biomarker assessment, inclusion of multiple biomarker classes, and representation of diverse PCI settings ranging from elective to high-risk cohorts such as primary and chronic total occlusion interventions. The integration of both prognostic and pathophysiologic perspectives enhances the interpretive depth and allows for a more comprehensive understanding of biomarker behavior following PCI. However, several limitations should be acknowledged. The included studies demonstrate considerable heterogeneity in biomarker timing, definitions of elevation, threshold selection, and reported clinical endpoints, which limit direct comparability and preclude quantitative synthesis. Additionally, most studies are observational in nature and subject to residual confounding, particularly related to baseline disease severity, procedural complexity, and comorbid conditions. Variability in assay types and lack of standardized protocols for serial measurement further constrain generalizability. Future research should focus on standardizing timing and definitions of dynamic biomarker assessment, establishing clinically meaningful delta thresholds, and developing integrated risk models that incorporate both static and temporal biomarker data. Prospective studies and randomized evaluations are needed to determine whether biomarker-guided strategies can improve clinical outcomes and inform decision-making pathways in patients undergoing PCI.

## Conclusions

In patients undergoing percutaneous coronary intervention, the prognostic utility of cardiac biomarkers extends beyond their absolute values and is more accurately interpreted within a temporal and clinical context. While baseline troponin remains a consistent predictor of outcomes and post-procedural elevations demonstrate variable, threshold-dependent significance, emerging evidence indicates that dynamic biomarker assessment, particularly with NT-proBNP, provides enhanced prognostic discrimination and closer alignment with underlying pathophysiologic processes. These findings support a shift from reliance on isolated measurements toward a more integrated approach that incorporates biomarker trajectories, biomarker class, and procedural context. Although current evidence does not yet warrant routine implementation of dynamic strategies in all settings, it highlights a meaningful opportunity to refine risk stratification and personalize post-PCI care. Future efforts should focus on standardizing dynamic assessment frameworks and validating their clinical utility within prospective, biomarker-guided models.
